# Vertical inhibition of the PI3K/Akt/mTOR pathway is synergistic in breast cancer

**DOI:** 10.1038/oncsis.2017.86

**Published:** 2017-10-09

**Authors:** S-U Woo, T Sangai, A Akcakanat, H Chen, C Wei, F Meric-Bernstam

**Affiliations:** 1 Department of Surgical Oncology, The University of Texas MD Anderson Cancer Center, Houston, TX, USA; 2 Department of Investigational Cancer Therapeutics, The University of Texas MD Anderson Cancer Center, Houston, TX, USA; 3 Department of Biostatistics, The University of Texas MD Anderson Cancer Center, Houston, TX, USA

## Abstract

Deregulation and activation of the phosphoinositide 3-kinase (PI3K)/Akt/mammalian (or mechanistic) target of rapamycin (mTOR) pathway have a major role in proliferation and cell survival in breast cancer. However, as single agents, mTOR inhibitors have had modest antitumor efficacy. In this study, we evaluated the effects of vertical inhibition of mTOR and Akt in breast cancer cell lines and xenografts. We assessed the effects of mTOR inhibitor rapamycin and Akt inhibitor MK-2206, given as single drugs or in combination, on cell signaling, cell proliferation and apoptosis in a panel of cancer cell lines *in vitro*. The antitumor efficacy was tested *in vivo*. We demonstrated that MK-2206 inhibited Akt phosphorylation, cell proliferation and apoptosis in a dose-dependent manner in breast cancer cell lines. Rapamycin inhibited S6 phosphorylation and cell proliferation, and resulted in lower levels of apoptosis induction. Furthermore, the combination treatment inhibited phosphorylation of Akt and S6, synergistically inhibited proliferation and induced apoptosis with a higher efficacy. *In vivo* combination inhibited tumor growth more than either agent alone. Our data suggest that a combination of Akt and mTOR inhibitors have greater antitumor activity in breast cancer cells, which may be a viable approach to treat patients.

## Introduction

The phosphoinositide 3-kinase (PI3K)/Akt/mammalian (or mechanistic) target of rapamycin (mTOR) pathway regulates cell growth, protein translation, autophagy, metabolism and cell survival. Deregulation and activation of this pathway is associated with tumorigenesis and cancer progression. One or more PI3K pathway components were altered in 38% of cancer patients.^1^ Phosphatase and tensin homolog (PTEN) loss (30% by immunohistochemistry), *PIK3CA* (13%), *PTEN* (6%) and *AKT1* (1%) mutations are common and more frequently observed with hormone receptor overexpression (androgen, progesterone or estrogen receptor) or human epidermal growth factor receptor 2 (HER2) amplification.^1^ *In vitro* data suggest that tumors with a low level PTEN expression or a mutant *PIK3CA* depend on Akt for oncogenic signaling, such as elevation of phosphorylated Akt levels, is frequently observed in breast cancer and indicate poor prognosis.^2^ Furthermore, in breast cancer, PI3K/Akt/mTOR pathway is associated with resistance to endocrine therapy, HER2-directed therapy and cytotoxic therapy.^3, 4^

Rapamycin is an allosteric inhibitor of mTOR. Everolimus, a rapamycin analog (rapalog), is approved by US Food and Drug Administration in combination with exemestane to treat postmenopausal women with advanced hormone receptor positive, HER2-negative breast cancer. Rapalogs have also been approved for the treatment of neuroendocrine tumors, renal cell carcinoma and subependymal giant cell astrocytoma associated with tuberous sclerosis. Mostly, therapeutic effects were observed in a subset of patients that was leading to disease stabilization rather than tumor regression. The efficacy is likely limited by feedback regulations within the pathway and crosstalk with other pathways. Under normal conditions, feedback regulation from mTOR complex 1 (mTORC1)/S6K1 to insulin receptor substrate-1 (IRS-1) attenuates cell growth signals.^5, 6, 7^ In addition, S6K1 phosphorylates rictor and reduces mTOC2 signaling.^8^ In some cancer cells treated with rapalogs, these two feedback loops can enhance PI3K/Akt signaling. The effect of this Akt activation remains unclear; it is proposed as a marker of resistance but also as an indicator of rapamycin activity, thus a marker of sensitivity.^9, 10^ Thus, combination treatments with chemotherapeutic or small molecule inhibitor agents are advised.

MK-2206 (Merck Oncology) is an allosteric inhibitor of Akt. It predominantly inhibits Akt1/2 in nanomolar range and has reduced potency against Akt3.^11^ It has potent anti-proliferative activity in cell lines with a *PIK3CA* activating mutation, inactivation of PTEN and amplification or mutation of Akt.^11^ Inhibition of phosphorylation at both Akt T308 and S473 residues results in inhibition of Akt signaling and cell cycle progression, while an increase in apoptosis.^12^ It has been shown to synergize with cytotoxic agents and pathway inhibitors *in vitro* and *in vivo*.^13, 14^ MK-2206 monotherapy demonstrated limited antitumor effect in phase II trials and currently it is being tested in combination with chemotherapeutics and small molecule inhibitors.^15^

To increase drug efficacy and overcome resistance, multiple key mediators of tumor survival signaling pathways are being targeted. First approach is to use two inhibitors targeting two pathways (parallel inhibition), such as PI3K/Akt/mTOR and Raf/MEK/Erk pathways.^16^ A second method is targeting a pathway at two points (vertical inhibition) by using a dual inhibitor or two inhibitors. There are several dual inhibitors now, and to overcome negative feedback loop Akt activation, the vertical inhibition of PI3K/Akt/mTOR pathway has been studied.^17^ In this study, we evaluated the antitumor growth effect of rapamycin and MK-2206 combination *in vitro* and *in vivo*. Our objective was to determine whether this combination would be synergistic. We tested the effect of this combination on cell proliferation, cell cycle progression and apoptosis using breast cancer cell lines.

## Results

### MK-2206 inhibits Akt signaling but does not inhibit S6K axis in every cell line

**Figure 1:**
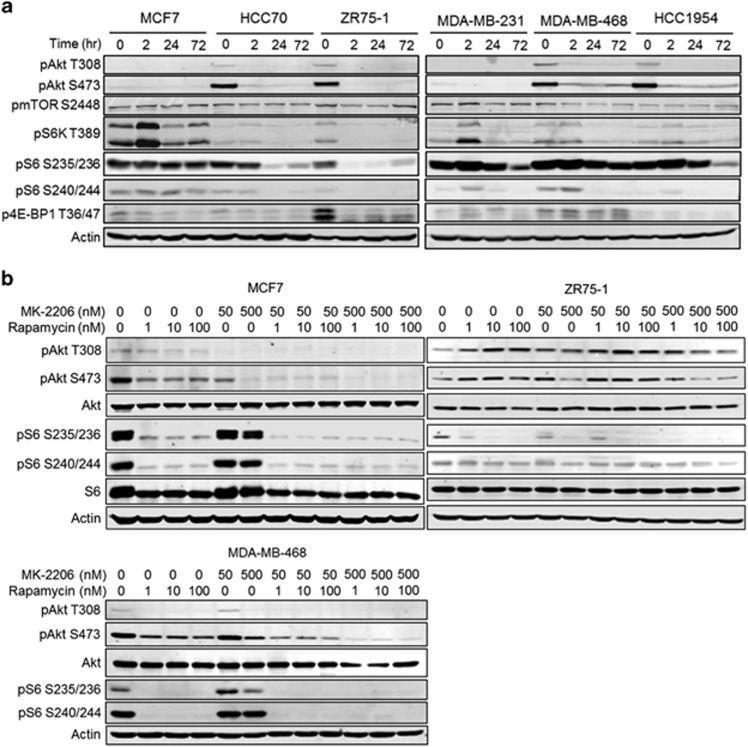
MK-2206 and rapamycin regulate phosphorylation of Akt/mTOR pathway proteins. (**a**) A panel of breast cancer cell lines was treated with MK-2206 500 nM for 2, 24 or 72 h. The changes in phosphorylation status of Akt, mTOR, S6K, S6 and 4E-BP1 in time was analyzed by western blotting using phospho-specific antibodies. (**b)** Three breast cancer cell lines were treated with various doses of MK-2206, rapamycin and their combination for 72 h. Regulation of Akt and mTOR signaling was demonstrated by western blotting. All experiments were repeated three times.

We treated six breast cancer cell lines of different subtypes and genomic backgrounds with MK-2206 at concentration of 500 nM and harvested the cells after 2, 24 and 72 h (Figure 1a). Akt phosphorylation was inhibited at 2 h and this inhibition was sustained for at least 72 h. S6K and S6 phosphorylation was decreased in all cell lines. However, a biphasic pattern was observed in in HCC1954, MCF7 and MDA-MB-231, S6K and S6 phosphorylation was initially increased at 2 h and decreased in late time points. In ZR75-1 cell line, 4E-BP1 phosphorylation was inhibited at 2 h but in MDA-MB-468 cell line, there was no inhibition. The other cell lines did not have a strong phospho-4E-BP1 expression and it was difficult to assess its regulation.

Next, we tested MK-2206 and rapamycin in combination. We selected three rapamycin-sensitive cell lines^10^ and treated with various doses of both drugs for 72 h (Figure 1b). S6 and Akt were selected as the markers to confirm rapamycin and MK-2206 activity, respectively. High-dose MK-2206 and rapamycin combination resulted in almost complete inhibition of phosphorylation of both S6 and Akt in all three cell lines. We did not observe a significant decrease in Akt phosphorylation in ZR75-1 cell line with low-dose MK-2206 treatment alone, particularly at Akt T308, and only the combination was effective.

### MK-2206 and rapamycin synergistically inhibit breast cancer cell growth

**Figure 2:**
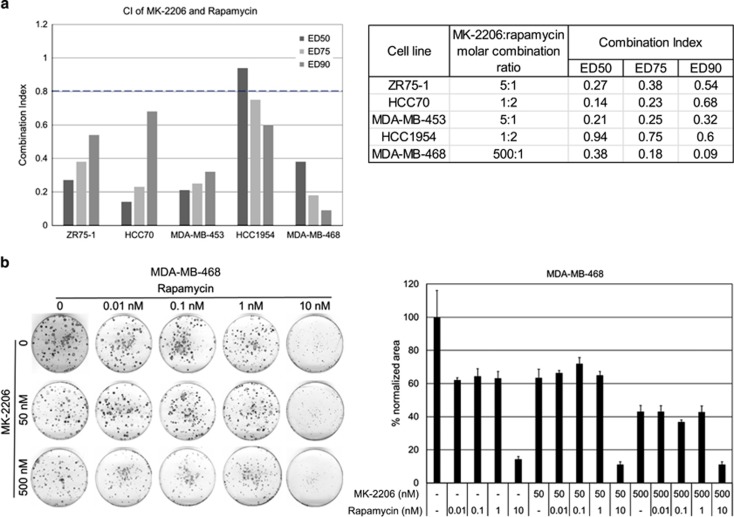
Combining MK-2206 and rapamycin synergistically suppress growth *in vitro*. (**a**) Five breast cancer cell lines were treated with vehicle, MK-2206, rapamycin or a combination of MK-2206 and rapamycin for 96 h in triplicates. The effect on cell growth was assessed by SRB assay and CI values were calculated. The chart displays CI of MK-2206 and rapamycin combination at ED50, ED75 and ED90. CI<0.8 represents synergy. (**b**) MDA-MB-468 cell lines were treated with vehicle, and increasing concentrations of MK-2206, rapamycin or a combination MK-2206 and rapamycin in triplicates. The colonies were fixed and stained. The surface area occupied by colonies is normalized to vehicle control. One of the three experiment in triplicate cultures was shown as mean±s.e.m.

In a panel of six breast cancer cell lines, combination index (CI) was calculated based on dose-effect levels of median-effect plots of MK-2206 alone, rapamycin alone and their combination at fixed molar ratios. CI values were consistently <1 for five cell lines indicating synergy (Figure 2a). HCC1954 and MDA-MB-468 cell lines are resistant to MK-2206,^12^ and, at effective dose 90 (ED90) there was more synergy compared with ED50 and ED75. This was the opposite for the other three sensitive cell lines, there was more synergy at ED50. We were not able to calculate CI for MDA-MB-231 cell line. As reported previously, this cell line was resistant to both MK-2206 and rapamycin.^10, 12^

We performed colony formation assay to measure growth inhibition because of treatment with MK-2206, rapamycin and their combination (Figure 2b). Quantitation and normalization of the colony formation assay was performed to provide and objective measurement of growth inhibition. Compared with control, all treatment groups significantly inhibited growth (*P*-values ranging from <0.05 to <0.0001). However, combinations at various doses did not show a reduction of growth compared with single-agent treatments.

### MK-2206 induces apoptosis

**Figure 3:**
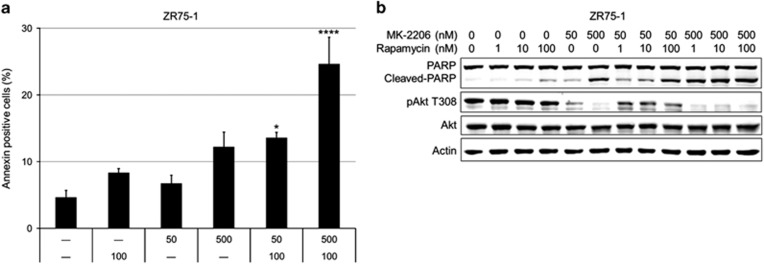
Combining MK-2206 and rapamycin synergistically increase apoptosis *in vitro*. (**a**) ZR75-1 cell line was treated with vehicle, MK-2206, rapamycin or a combination of MK-2206 and rapamycin for 72 h in triplicates. The percentages of annexin V-positive cells were determined by flow cytometry and compared. Average of three experiments is represented (mean±s.e.m.; *P<0.05; ****P<0.0001). (**b**) ZR75-1 cells were treated with vehicle, MK-2206, rapamycin or a combination of MK-2206 and rapamycin for 72 h. Western blotting was conducted to assess Akt phosphorylation and apoptosis. All experiments were repeated three times.

ZR75-1 cell line was treated with vehicle, rapamycin, MK-2206 and their combination for 72 h. The percentages of annexin V-positive cells were determined by flow cytometry (Figure 3a). Both low- and high-dose MK-2206 combined with rapamycin caused a significant increase in apoptotic cell death (at 50 nM, *P*<0.05, at 500 nM, *P*<0.0001). Compared with high-dose MK-2206 alone, high-dose MK-2206 combination with rapamycin doubled the percentage of apoptotic cells (mean±s.e.m.: MK-2206, 12.2±2.0; combination, 24.6±4.0; *P*<0.001). Western blotting of the PARP cleavage in response to MK-2206, rapamycin and combination treatment for 72 h is shown in Figure 3b. PARP cleavage clearly increased with high-dose MK-2206 treatment. The combination with rapamycin enhanced the PARP cleavage observed with low-dose MK-2206. Under the same treatment conditions, we did not observe apoptosis in MDA-MB-468 cell line (data not shown).

### Combination of MK-2206 and rapamycin inhibits tumor growth *in vivo*

**Figure 4:**
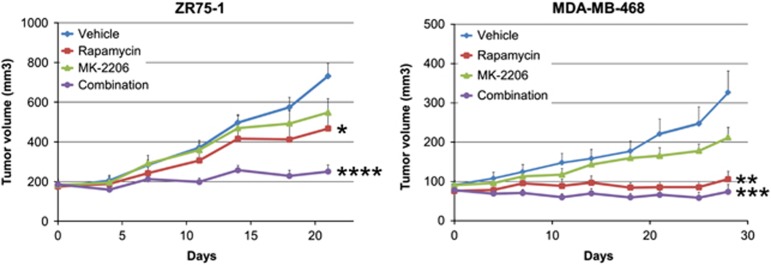
Combining MK-2206 and rapamycin synergistically suppress growth *in vivo*. Mice bearing ZR75-1 or MDA-MB-468 xenografts were treated with vehicle, MK-2206 240 mg/kg, rapamycin 10 mg/kg, or a combination of MK-2206 and rapamycin at the same doses once a week. Treatment group tumor volumes were compared with control at the last day of the experiment. Data were represented as mean±s.e.m. (*P<0.05; **P<0.01; ***P<0.001; ****P<0.0001).

Tumor volumes of four groups were compared at the final day of treatment (Figure 4). In both ZR75-1 and MDA-MB-468 cell line xenografts, MK-2206 alone inhibited tumor growth; however, this inhibition did not reach significance level of 0.05. Rapamycin alone was more effective compared with MK-2206 (vehicle vs rapamycin, ZR75-1 *P*<0.05 and MDA-MB-468 *P*<0.01) and the greatest inhibition of tumor growth was delivered by the MK-2206 and rapamycin combination treatment (vehicle vs combination, ZR75-1 *P*<0.0001 and MDA-MB-468 *P*<0.001).

### Functional proteomics demonstrated inhibition of Akt/mTOR pathway

The reverse phase proteomics array (RPPA) core facility antibody panel is enriched in PI3K/Akt/mTOR pathway proteins. MDA-MB-468 cell line has *PTEN* loss,^18^ high EGFR expression^19^ and activated PI3K/Akt/mTOR pathway. ZR75-1 has a *PTEN* (L108R) mutation^20^ with very weak PTEN expression and high levels of pAkt.^18^ Regardless of both cell lines having Akt/mTOR pathway activation, they showed alteration of different markers (Supplementary Figure 1). The combination treatment arm showed that pAkt, its downstream target pGSK 3, and mTOR downstream target p4E-BP1 were inhibited. Notch 1 and tuberin were downregulated, and AXL and collagen VI were upregulated in both cell line xenografts.

## Discussion

Activation of PI3K/Akt/mTOR pathway is a central event in many types of cancer and represents a promising target for new treatment strategies. However, there is modest antitumor activity of single-agent therapies suggesting that there is a need for drug combinations to induce superior clinical responses. In this study, we investigated the vertical targeting of PI3K/Akt/mTOR pathway in breast cancer cells. After MK-2206 and rapamycin treatment, key downstream proteins within Akt/mTOR pathway were dephosphorylated in breast cancer cell lines, including Akt, mTOR, S6 and 4E-BP1. This combination produced a synergistic effect against breast cancer cell proliferation *in vitro*. Furthermore, this combination induced apoptosis *in vitro* and inhibited tumor growth more compared with single drug groups in animal models. Our drug dosing schedule in mice was similar to the schedule in humans and we did not observe any signs of toxicity. The data support dual targeting of PI3K/Akt/mTOR pathway in cancer treatment.

Western blotting showed that rapamycin and MK-2206 combination blocked Akt/mTOR signaling completely. However, MK-2206 as single drug activated S6K and S6 at 2-h time point. Biphasic phosphorylation of S6 was also reported after rapamycin treatment.^21^ To our knowledge, there is no clear explanation of this event and this finding indicates complexity of S6K/S6 phosphorylation.

In many studies, pS6 is used as a marker of mTOR activity, however, in our study RPPA did not show a decrease in pS6 levels. We collected xenograft tumors 24 h later following the last injection of drugs. It is possible that this period was too long to detect alterations in phosphorylation status of target proteins. Interestingly, combination treatment regulated expression levels of four proteins in both cell line xenografts. Of the two upregulated proteins, collagen VI expression was reported to be upregulated in breast cancer promoting tumor progression and metastasis.^22^ AXL overexpression is poor prognostic and contributes to the functional skewing of macrophage functions in triple-negative breast cancer (TNBC).^23^ Vimentin-positive and AXL high-expressing TNBCs have shorter recurrence-free and overall survival.^24^ Of the two downregulated proteins, in a meta-analysis, Notch 1 is found to be overexpressed in basal subtype of breast cancer and associated with transition from ductal carcinoma *in situ* to invasive cancer.^25^ Tuberin expression is low in breast cancer tumors compared with normal tissues, and patients with recurrence and mortality have been reported to have significantly lower levels compared with those who remained disease free.^26^ Interestingly, three of four regulated proteins are associated with poor prognosis. We need to validate these results in a larger number of preclinical models to understand the underlying mechanisms. These alterations may represent adaptive responses to facilitate survival upon treatment with PI3K/Akt/mTOR inhibitors. This opens up the possibility that targeting these survival pathways in combination, may further enhance antitumor efficacy.

Elevated phospho-Akt levels predicted increased sensitivity to mTOR inhibition *in vitro*.^10^ In a rapamycin-resistant diffuse large B-cell lymphoma model, a gene expression profile was generated.^27^ This model identified lymphoma subsets that were resistant to mTOR inhibitor therapy and mapped compounds targeting Akt as a way of reversing mTOR inhibitor resistance.^27^ MK-2206 and a rapalog combination was synergistic in other types of malignancies such as B-precursor acute lymphoblastic leukemia,^28^ cholangiocarcinoma,^29^ hepatocellular carcinoma^30, 31^ and neuroblastoma^32^ *in vitro* and *in vivo*, in multiple myeloma,^33^ gastric cancer^34^ and thyroid cancer^35^ *in vitro*. Using a genetically engineered mouse model of castration-resistant prostate cancer model, Floc’h *et al.*^36^ tested combination of MK-2206 and a rapalog ridaforolimus (MK-8669). They reported that the feedback loop activation was observed in human prostate cancer cell lines but not *in vivo* model, and suggested that different factors, such as mTORC2 activation of Akt, might contribute to the enhanced efficacy.^36^ In an MK-2206-induced acquired resistance model of neuroblastoma cell lines, MK-2206-resistant cells showed increased sensitivity to mTOR (AZD8805) and PDK1 (GSK2334470) inhibitors.^37^ These cells were characterized by low Akt phosphorylation and elevated PDK1/mTOR/S6K activity, and inhibition of PDK1/mTOR/S6K signaling was suggested as a way to overcome the MK-2206 resistance. Recently, Mi *et al.*^38^ stated that sustained 4E-BP1 phosphorylation resulted in rapamycin resistance and effective inhibition of PRAS40 phosphorylation was required to overcome this resistance. Dephosphorylation of PRAS40 represses Akt/mTORC1 regulated 4E-BP1 phosphorylation. To confirm this statement, we selected ZR75-1 cell line, where baseline 4E-BP1 phosphorylation was the highest (Figure 1a) and tested three phosphorylation sites, T36/47, S65 and T70. A significant decrease in PRAS40 phosphorylation was not observed (data not shown). However, combination treatment was more effective in suppressing 4E-BP1 phosphorylation, suggesting that combination therapy may more effectively inhibit cap-dependent translation.

The need for combining MK-2206 with other targeting compounds to treat breast cancer is emphasized in two recently reported studies. In a patient-derived xenograft model of basal-like breast cancer, there was synergistic effect of MK-2206 and ridaforolimus on tumor growth and cell proliferation.^39^ PTEN knockdown increased this synergy suggesting a rationale to use this combination in basal-like breast cancer with PTEN loss. A phase I study of MK-2206 and ridaforolimus in advanced cancers was recently reported. The cohort was enriched in breast cancer patients with low RAS gene signature (a score derived from expression of 147 transcripts) or ER+ patients with high Ki-67 index (high proliferation).^40^ Partial and complete response were observed in 12.5% and 14.3% of the patients, respectively. The combination had signal of activity in heavily treated breast cancer patients with exhibiting PI3K pathway dependence. Precinically, other Akt inhibitors were also tested in combination with rapalogs. In human breast cancer cell lines, rapamycin and A-443654 combination induced G2-M arrest and apoptosis.^41^ Use of perifosine and temsirolimus to treat mice with gliomas resulted in decreased Akt and mTOR signaling, decreased proliferation and increased cell death.^42^ All listed preclinical studies taken together demonstrate that inhibition of multiple components of Akt/mTOR pathway is superior to inhibition of a single target.

The advantage of dual targeting of mitogen-activated protein kinase (MAPK) pathway has been clearly demonstrated in melanoma. MEK inhibitors significantly enhanced antitumor efficacy in when given in combination with BRAF inhibitors for BRAF V600E mutant melanoma both in preclinical models and in clinical trials, leading to the FDA approval of dabrafenib and trametinib,^43, 44, 45^ as well as vemurafenib with cobimetinib.^46^ Further, dual pathway inhibition decreased the toxicity profile, especially by decreasing development of cutaneous malignancies.^45^ In contrast, dual inhibition of PI3K and mTOR with dual inhibitors has been more challenging because of the toxicity profile of these agents. Similarly, Akt inhibitors and mTOR inhibitors would be expected to have overlapping toxicity. Indeed, in phase I the combination of ridaforolimus and MK-2206 was considered tolerable but several adverse events were common, such as rash (44%), stomatitis (39%), diarrhea (28%) and decreased appetite (28%).^40^ Thus, further work is needed to identify optimal agents, dose and schedule for vertical inhibition of PI3K/Akt/mTOR signaling with the combination of Akt inhibitor and mTOR inhibitor. There are three more ongoing phase I trials giving a combination of PI3K and mTOR inhibitors, and there are no results yet.^47, 48, 49^

In summary, considering that a major part of human breast cancers have activation of PI3K/Akt/mTOR pathway, targeting Akt and mTOR together in the treatment of this disease may enhance antitumor efficacy. Further studies are needed to determine the role of this therapeutic approach in different molecular subtypes and the clinical feasibility of this approach.

## Materials and methods

### Cell lines, cultures and reagents

HCC70, HCC1954, MCF7, MDA-MB-231, MDA-MB-453, MDA-MB-468 and ZR75-1 breast cancer cell lines were obtained from American Tissue Culture Collection (Manassas, VA, USA). All cell lines were cultured in Dulbecco’s modified Eagle’s medium/F12 supplemented with 10% fetal bovine serum at 37 °C and humidified in 5% CO_2_.

Captisol, dimethyl sulphoxide (DMSO), MK-2206 and rapamycin were purchased from CYDEX Pharmaceuticals (Lenexa, KS, USA), Sigma Chemical Company (St Louis, MO, USA), Selleckchem (Houston, TX, USA) and LC Laboratories (Woburn, MA, USA), respectively. For *in vitro* experiments, stock solutions of rapamycin and MK-2206 were prepared in DMSO at 10 mM. For *in vivo* experiments, stock solution of rapamycin was prepared in DMSO at 10 mg/ml, whereas MK-2206 was formulated in a 30% (w/v) Captisol solution.

### Cell proliferation assay

Cells were plated at 1500–2000 cells per well in 96-well plates and allowed to attach overnight. Cells were treated with increasing doses of MK-2206, rapamycin or a combination of MK-2206 and rapamycin for 4 days. Cell proliferation was assessed by sulforhodamine B (SRB) assay.^50^ The median inhibitory concentration (IC50) and CI were determined from dose-response curves for 4 days treatment by CalcuSyn (Biosoft, UK). CI values are presented at ED50, ED75 and ED90, to inhibit growth at 50%, 75% and 90%, respectively. CI indicates synergy (<0.8), additive (0.8–1.2), and antagonism (>1.2).

### Colony formation assay

MDA-MB-468 cells were plated at 2000 cells per plate in 6-cm plates and allowed to attach overnight. Cells were treated with increasing doses of MK-2206, rapamycin or a combination of MK-2206 and rapamycin once a week. Colony formation was assessed by crystal violet staining. Plates were scanned and colonies were counted using the ImageJ software.^51^ Relative colony area was determined by normalizing colony area of each plate to the average colony area of the control.

### Annexin V assay

Cells were treated for 72 h and apoptosis was identified by Annexin V Apoptosis kit (Roche, Indianapolis, IN, USA) according to the manufacturer’s protocol in the MD Anderson Flow Cytometry and Cellular Imaging Core Facility. Labeled cells were analyzed by flow cytometry and FlowJo (FlowJo, LLC, Ashland, OR, USA).

### Western blotting

Cultured cells were washed in cold PBS and lysed in buffer containing 62.5 mM Tris-HCl, pH 6.8, 2% sodium dodecyl sulfate and 25% glycerol. Protein lysates were separated by sodium dodecyl sulfate–polyacrylamide gel electrophoresis and transferred to 0.2 μm nitrocellulose membrane. After blocking with 0.1% casein, membranes were probed with antibodies to p4E-BP1 T36/47 (#9459), Akt (#9272), pAkt T308 (#13038), pAkt S473 (#9271), pmTOR S2448 (#2983), PARP (#9542), S6 (#2217), pS6 S235/236 (#4858), pS6 S240/244 (#2215), pS6K T389 (#9234) (Cell Signaling Technologies, Boston, MA, USA) and actin (#A5441) (Sigma-Aldrich Co., LLC, St Louis, MO, USA). The signals were visualized by Odyssey infrared imaging system (Li-Cor Biosciences, Lincoln, NE, USA).

### Reverse phase proteomics array

RPPA was done in the MD Anderson Cancer Center Functional Proteomics RPPA Core Facility as described previously.^10^ Proteomic profiles of MK-2206, rapamycin and combination treatment xenografts were compared with the vehicle. Relative expression values of proteins were in log2. The median polish normalized RPPA data set consisted of 285 proteins.

### *In vivo* studies

All animal experiments were approved by the MD Anderson Animal Care and Use Committee following Public Health Service Animal Welfare Assurance #A334301. ZR75-1 (1 × 10^7^) and MDA-MB-468 (5 × 10^6^) cells were inoculated in the mammary fat pads of female nu/nu mice (Department of Experimental Radiology, MD Anderson). Mice were randomized into combination vehicle control, MK-2206 240 mg/kg, rapamycin 10 mg/kg, and MK-2206 and rapamycin combination therapy groups. MK-2206 and rapamycin were administered by oral gavage and intraperitoneal injection, respectively. There were seven mice in each group and all treatments were given weekly. Tumor volumes were calculated as previously described.^52^ Mice were killed 24 h after the last treatment. Tumors were snap-frozen for further analysis.

### Statistical analysis

For *in vitro* and *in vivo* studies, comparisons of all groups were carried out by one-way analysis of variance (ANOVA) with Tukey’s test for multiple comparisons using (GraphPad Prism software, Inc., La Jolla, CA, USA). Xenograft tumor volumes were compared at the final day of treatment. Data were presented as means±s.e. A two-sided *P*-value <0.05 was considered significant.

For each cell line xenograft, one-way ANOVA was used to assess the differences in protein expression levels between treatment groups on a marker-by-marker basis. An unadjusted overall F-test *P*-value of<0.05 and an unadjusted pairwise comparison *P*-value of <0.05 were used to determine if there is any significant difference among any of the means by treatment group. The contrasts of the desired comparisons between treatment groups were performed using the ‘multcomp’ R package (https://cran.r-project.org/web/packages/multcomp). To account for multiple testing, the false discovery rates of the overall F-test of the model was estimated using the Benjamini–Hochberg method.^53^

## Supplementary Material

Supplementary Figure Legend

Supplementary Figure s1
