# Supplementary material for: Vertical inhibition of the PI3K/Akt/mTOR pathway is synergistic in breast cancer
Source: Oncogenesis. 2017 Oct 9;6(10):e385–. doi: 10.1038/oncsis.2017.86 (PMC5668884; doi:10.1038/oncsis.2017.86)
Supplement: Supplementary Figure Legend [file oncsis201786x1.docx]

**Supplementary Figures**

Supplementary Figure 1: Differentially expressed proteins in MDA-MB-468 and ZR75-1 xenograft models. An unadjusted overall F-test p-value of < 0.05 and unadjusted pairwise comparison p-value of < 0.05 was regarded as significant.
