# Supplementary figures and images for: Vertical inhibition of the PI3K/Akt/mTOR pathway is synergistic in breast cancer
Source: Oncogenesis. 2017 Oct 9;6(10):e385–. doi: 10.1038/oncsis.2017.86 (PMC5668884; doi:10.1038/oncsis.2017.86)

Supplementary  
Figure 1

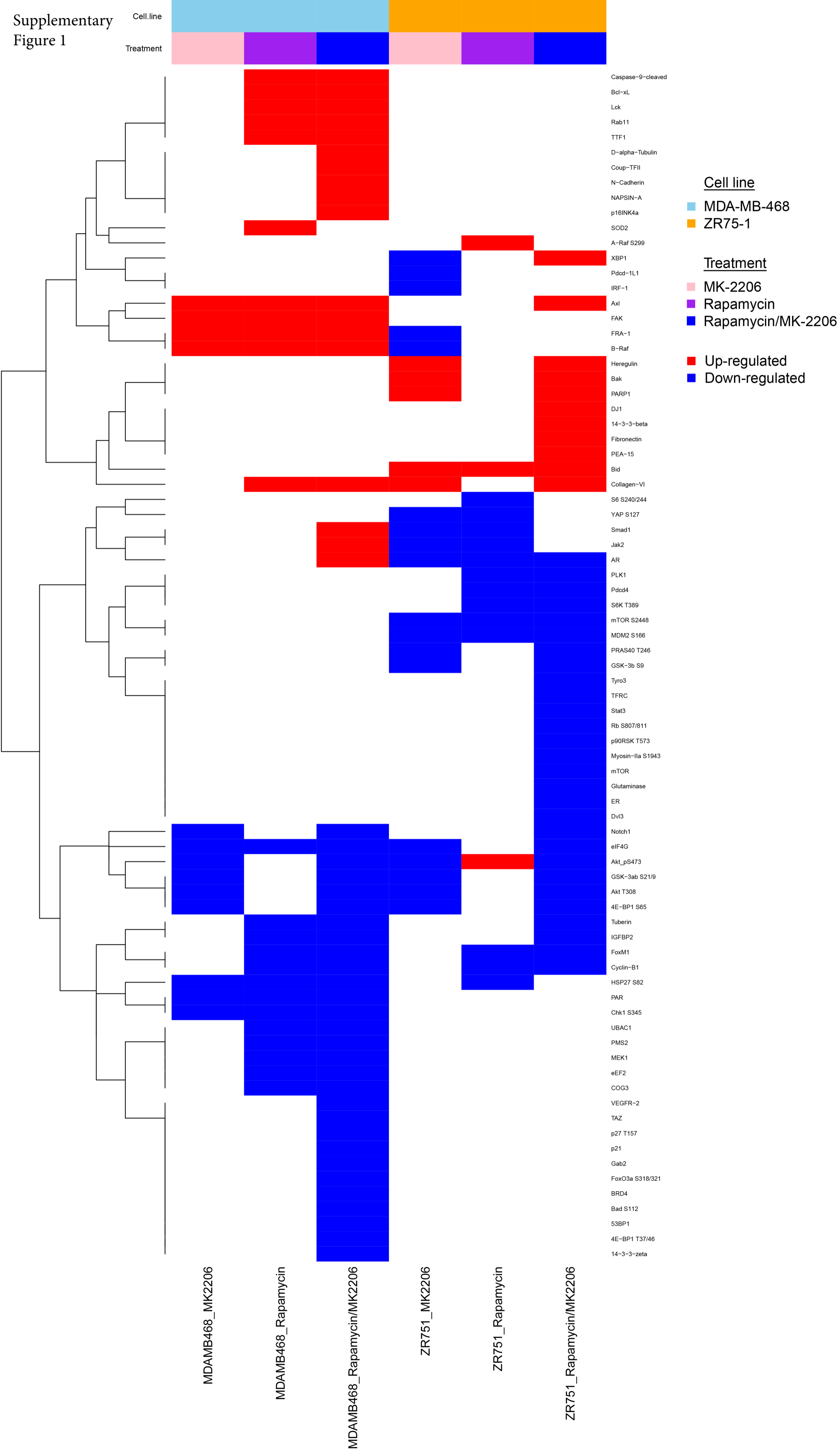

Supplement: Supplementary Figure s1 [file oncsis201786x2.pdf]
